# Comparison of two ASC-derived therapeutics in an in vitro OA model: secretome versus extracellular vesicles

**DOI:** 10.1186/s13287-020-02035-5

**Published:** 2020-12-03

**Authors:** Chiara Giannasi, Stefania Niada, Cinzia Magagnotti, Enrico Ragni, Annapaola Andolfo, Anna Teresa Brini

**Affiliations:** 1 Laboratorio di Applicazioni Biotecnologiche, IRCCS Istituto Ortopedico Galeazzi, Milan, Italy; 2 Proteomics and Metabolomics Facility (ProMeFa), IRCCS San Raffaele Scientific Institute, Milan, Italy; 3 Laboratorio di Biotecnologie Applicate all’Ortopedia, IRCCS Istituto Ortopedico Galeazzi, Milan, Italy; 4 Department of Biomedical, Surgical and Dental Sciences, Università degli Studi di Milano, Milan, Italy

**Keywords:** Adipose-derived stem/stromal cells, Secretome, Extracellular vesicles, Chondrocytes, Osteoarthritis, Hypertrophy, MMP, PGE2

## Abstract

**Background:**

In the last years, several clinical trials have proved the safety and efficacy of adipose-derived stem/stromal cells (ASC) in contrasting osteoarthritis (OA). Since ASC act mainly through paracrine mechanisms, their secretome (conditioned medium, CM) represents a promising therapeutic alternative. ASC-CM is a complex cocktail of proteins, nucleic acids, and lipids released as soluble factors and/or conveyed into extracellular vesicles (EV). Here, we investigate its therapeutic potential in an in vitro model of OA.

**Methods:**

Human articular chondrocytes (CH) were induced towards an OA phenotype by 10 ng/ml TNFα in the presence of either ASC-CM or EV, both deriving from 5 × 10^5^ cells, to evaluate the effect on hypertrophic, catabolic, and inflammatory markers.

**Results:**

Given the same number of donor cells, our data reveal a higher therapeutic potential of ASC-CM compared to EV alone that was confirmed by its enrichment in chondroprotective factors among which TIMP-1 and -2 stand out. In details, only ASC-CM significantly decreased MMP activity (22% and 29% after 3 and 6 days) and PGE2 expression (up to 40% at day 6) boosted by the inflammatory cytokine. Conversely, both treatments down-modulated of ~ 30% the hypertrophic marker COL10A1.

**Conclusions:**

These biological and molecular evidences of ASC-CM beneficial action on CH with an induced OA phenotype may lay the basis for its future clinical translation as a cell-free therapeutic in the management of OA.

**Supplementary information:**

The online version contains supplementary material available at 10.1186/s13287-020-02035-5.

## Background

Osteoarthritis (OA) is an age-related disease that affects millions of people worldwide, representing a leading cause of locomotor disability and therefore entailing high socio-economic costs [1]. Its pathogenesis is complex and engages different tissues. However, despite the proved involvement of subchondral bone and synovium during the degenerative process, the impairment of both the structure and the function of articular cartilage is still recognized as one of the earliest disease causing events [2, 3].

Currently, OA cannot be reversed pharmacologically, but treatments can help relieve its symptoms. The medications most commonly used in OA treatment are intra-articular corticosteroids, topical and oral non-steroidal anti-inflammatory drugs (NSAID), duloxetine, and acetaminophen, often accompanied by physiotherapy and life style modification [4, 5]. In the worst cases, when conservative approaches fail, arthroplasty remains the only option. Although the available treatments improve the quality of life in OA patients by reducing pain and promoting joint mobility, the need to achieve adequate tissue regeneration and to develop drugs able to modify the course of the disease (disease-modifying anti-OA drugs, DMOAD) is still unmet. In this context, orthobiologics are emerging as alternative therapeutic tools, thanks to their regenerative potential and cost-effectiveness [6, 7]. These approaches include intra-articular injection of platelet-rich plasma (PRP) and biografts, such as autologous chondrocyte implantation (ACI), bone marrow concentrate (BMC), and adipose-derived stem/stromal cell (ASC) therapy. The latter techniques rely on the presence, in both bone marrow and fat, of progenitor cells called mesenchymal stem/stromal cells (MSC). In response to various stimuli, MSC can differentiate into specialized cell types and/or behave as “signaling” cells, able to pour into the microenvironment several mediators, such as nucleic acids, proteins, and lipids, that orchestrate the regenerative process by modulating the immune system and recruiting specialized effectors (e.g., mast cells and T lymphocytes). In the last years, in vitro [8–10] and in vivo [11–13] studies have proved the therapeutic potential of MSC in counteracting cartilage damage and, to date, more than 100 clinical trials have evaluated/are assessing the safety and efficacy of MSC intra-articular injection in OA patients (http://www.clinicaltrials.gov). Since nowadays it is widely accepted that MSC action is largely mediated by paracrine mechanisms [14], the scientific interest has shifted towards the study of their secretome, the conditioned medium (MSC-CM). Indeed, cell secretome is a cocktail of soluble factors and extracellular vesicles (EV) with a promising potential in regenerative applications. EV are particles naturally released from the cell that are delimited by a lipid bilayer and may be of both endosomal origin or plasma membrane-derived [15]. Since a consensus has not emerged on specific markers of EV subtypes yet, the recent nomenclature established by the International Society for Extracellular Vesicles (ISEV) divides EV into small (< 200 nm) and large (> 200 nm) particles, previously called exosomes and microvesicles based on their endosomal or plasma membrane origin [16]. Two works have recently reviewed the available evidence of MSC-CM therapeutic action on cartilage, subchondral bone and synovium [17, 18]. Among other MSC sources, adipose tissue presents several advantages in terms of harvesting procedure, cell isolation, and expansion [19]. ASC efficacy and safety have been largely studied, both in vitro and in vivo, and confirmed by clinical trials [20, 21]. Moreover, ASC therapy in the treatment of COVID-19 disease has recently shown promising outcomes [22, 23]. In recent years, our group investigated and characterized ASC-CM content in terms of both soluble factors [24] and vesicular components [25, 26]. Furthermore, we evaluated ASC-CM effects in vitro on a model of human articular chondrocytes (CH) induced towards an OA-like phenotype by the inflammatory cytokine TNFα [27]. In our previous study, we proved that ASC-CM contains high levels of chondroprotective factors and exerts short-term anti-hypertrophic and anti-catabolic effects on TNFα-treated CH, confirming the potential of this cell-free approach in the management of OA. The present work aims at disclosing which components of ASC secretome play the major role in its beneficial action, by comparing the effects of ASC-CM and ASC-EV deriving from 5 × 10^5^ cells in the same OA *in vitro* model.

## Methods

Unless otherwise stated, reagents were purchased from Sigma-Aldrich, St. Louis, MO, USA.

### Cell cultures

Cell cultures were obtained from waste tissues collected at IRCCS Istituto Ortopedico Galeazzi upon Institutional Review Board approval. Written informed consent was obtained from all donors. In detail, ASC (1 male and 3 females; 43 ± 15 years old) and CH (4 males and 3 females, 64 ± 13 years old) were isolated from patients undergoing esthetic or prosthetic surgery, following well-established protocols [24, 27, 28]. Briefly, after mechanical fragmentation of the subcutaneous adipose tissue deriving from abdominoplasty surgery (*n* = 3) or abdominal liposuction (*n* = 1), ASC were isolated by enzymatic digestion with 0.75 mg/ml type I Collagenase (Worthington Biochemical Corporation, Lakewood, NJ, USA) for 30 min and filtering of the stromal vascular fraction through a 100-μm cell strainer (Corning Incorporated, Corning, NY, USA) [29]. All ASC donors were normal-weight subjects (BMI < 30, no documented diagnosis of obesity). CH derived from the femoral head of OA patients who underwent total hip replacement: only the areas of macroscopically healthy cartilage (white, shiny, elastic, and firm) were harvested through a scalpel and digested overnight at 37 °C with 1.5 mg/ml type II Collagenase (Worthington Biochemical Corporation, Lakewood, NJ, USA) [28, 30]. The areas characterized by irregular surface, discoloration or softening were never collected, even at the cost of losing the entire sample, in order to exclude any experimental bias linked to the use of strongly compromised cartilage. Cells were cultured in high glucose DMEM supplemented with 10% FBS (Euroclone, Pero, Italy), 2 mM L-glutamine, 50 U/ml penicillin, and 50 μg/ml streptomycin at 37 °C in a humidified atmosphere with 5% CO_2_. The culture medium was further implemented with 110 μg/ml sodium pyruvate for CH maintenance. Prior serum starvation for CM and EV production, ASC were characterized as previously described [31–34] and their features are summarized in Supplementary Table 1.

### CM and EV production

Conditioned medium was collected from ~ 90% confluent ASC (IV to VI passage) cultured for 72 h under starving conditions (absence of FBS), following optimized procedures [24]. Cells were monitored every day. No signs of cell suffering (e.g., detaching) was ever observed and cell viability was maintained for the whole starving duration (data not shown), consistently with a recent report by Petrenko et al. [35] After 72 h, conditioned media were collected and centrifuged at 2500×*g* for 15 min at 4 °C to remove dead cells, large apoptotic bodies, and debris. The supernatants were split in half to obtain coupled CM and EV samples, while donor cells were counted in order to correlate cell number to the appropriate treatment volumes. An aliquot of conditioned medium was centrifuged for 90 min at 4000×*g*, 4 °C, inside Amicon Ultra-15 Centrifugal Filter Devices with 3-kDa cut-off (Merck Millipore, Burlington, MA, USA), resulting in a 40–50-fold more concentrated final product. This procedure leads to a final product whose safety and efficacy have been already shown both in vitro [27] and in vivo [36]. In parallel, EV isolation was performed starting from naïve conditioned medium through differential centrifugation at 100,000×*g*, 4 °C [25]. Both final products were characterized as follows.

### Secretome characterization

#### Nanoparticle tracking analysis (NTA)

Coupled ASC-CM and ASC-EV samples were appropriately diluted in 0.22 μm triple-filtered PBS and analyzed by NanoSight NS300 (Malvern PANalytical, Salisbury, UK). For each measurement, 3 videos lasting 1 min were captured. All measurements matched the quality criteria of 20–120 particles/frame, concentration of 10^6^–4 × 10^9^ particles/ml and valid tracks > 20%. Upon capture, videos were analyzed by the in-build NanoSight Software NTA.

#### Cytofluorimetry

**Fig. 1:**
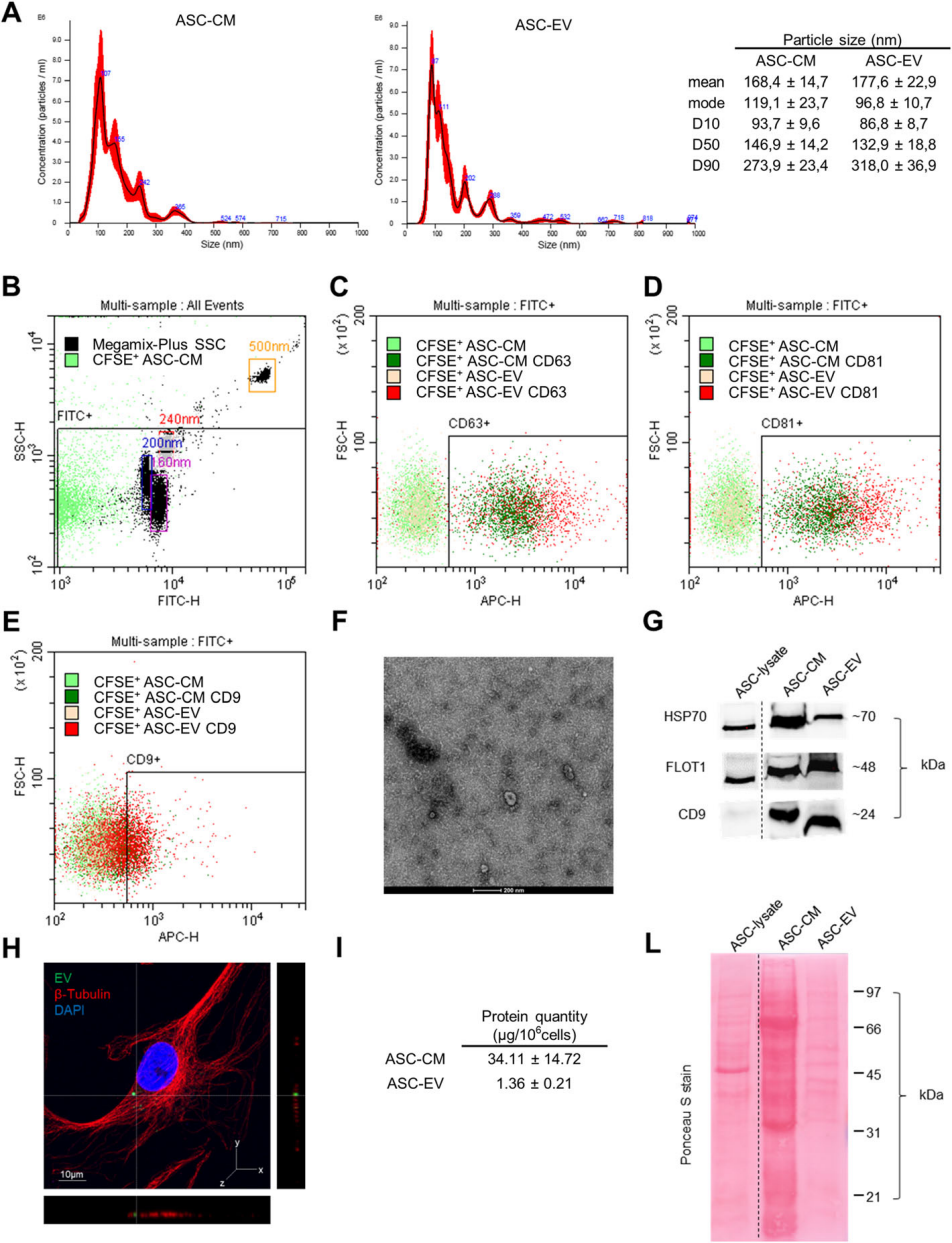
Characterization of ASC-CM and -EV. **a** Representative images of NTA referred to ASC-CM (left) and ASC-EV (right). The table shows the dimensional parameters of the samples expressed as mean ± SD of 6 NTA measurements. **b** Flow cytometer calibration with standard beads and CFSE^+^ ASC-CM. The FITC^+^ gate encloses the coordinates in SSC-H and FITC-H channels where to expect the events of interest. **c**–**e** CD63, CD81, and CD9 staining of representative CFSE^+^ ASC-CM and ASC-EV samples. **f** Transmission electron microscopy image showing the characteristic morphology of EV. The scale bar indicates 200 nm. **g** Representative Western blot of ASC-CM and EV lysates deriving from 10^6^ ASC. Cell lysate from 5 × 10^4^ ASC is shown as control. **h** Laser scanning confocal microscopy of CH treated with ASC^GFP+^-EV for 3 days. β-Tubulin was revealed with an Alexa Fluor® 568 conjugated antibody (red), nuclei were stained with DAPI (blue) (magnification × 63). The scale bar indicates 10 μm and the orthogonal views were obtained by Fiji software. **i** Total protein content per million ASC (μg/10^6^ cells). Data are shown as mean ± SD (*n* = 4). **l** Ponceau S staining of ASC-CM and -EV lysates from 10^6^ ASC. Cell lysate from 5 × 10^4^ ASC is also shown

Prior to cytofluorimetry analysis, ASC-CM and ASC-EV were appropriately diluted in 0.22 μm triple-filtered PBS and stained with the green fluorescent dye CFSE (carboxyfluorescein diacetate succinimidyl ester). In details, ASC-derived products were incubated with 20 nM CFSE for 1 h at 37 °C [37], then analyzed without any further washing. All data were obtained using a CytoFLEX flow cytometer (Beckman Coulter, Brea, CA, USA). At first, instrument calibration was set using Megamix-Plus SSC (Biocytex, Marseille, France), a reference bead mixture composed of FITC fluorescent spheres of heterogeneous dimensions (160 nm, 200 nm, 240 nm, and 500 nm). CFSE-positive (CFSE^+^) ASC-CM was run to set in SSC-H and FITC-H channels the region where to expect our events, accordingly to the coordinates given by the standardization beads (Fig. 1b). CFSE^+^ samples were then incubated for 20 min at 4 °C in the dark with APC-conjugated antibodies raised against CD9, CD63, and CD81 (BioLegend, San Diego, CA, USA, dilution 1:20) EV markers, as per well-documented protocols and general ISEV guidelines for positive EV characterization [16, 38], then 1:2 diluted in 0.22 μm triple-filtered PBS and acquired for 300 s at a low flow rate [39]. PBS-diluted antibodies and unlabeled samples were used as appropriate controls.

#### Transmission electron microscopy

PBS-resuspended EV were absorbed for 10 min on Formvar carbon-coated grids, and excess liquid was removed by a filter paper. Two percent uranyl acetate solution was used as negative stain for 10 min and excess of liquid was removed by a filter paper. The grid was dried at room temperature. Eventually, absorbed EV were examined with a TALOS L120C transmission electron microscope (Thermo Fisher Scientific, Waltham, MA, USA) at 120 kV.

#### Western blotting of secretome samples

For CM and EV samples, protein concentration was determined using Bio-Rad Protein Assay (Bio-Rad, Milan, Italy) following standard procedures. ASC-CM and -EV samples presented a total protein concentration of 0.49 ± 0.27 and 0.11 ± 0.04 μg/μl, respectively. For Western Blot analyses, specimens were lysed in Laemli buffer and analyzed as exhaustively described in [25, 26]. Briefly, proteins from CM and EV lysates deriving from 10^6^ASC (respectively 34.1 and 1.4 μg for ASC-CM and -EV) were resolved into 12% SDS-PAGE, transferred onto a nitrocellulose membrane, stained for 1 min with Ponceau S, and then probed for the expression of the typical EV markers HSP70 (ExoAb, System Biosciences, Palo Alto, CA, USA, dilution 1:1000), FLOT1 (BD Biosciences, San Jose, CA, 250 μg/ml, 1:500 diluted), and CD9 (ExoAb, System Biosciences, Palo Alto, CA, USA, dilution 1:1000) [16, 40]. Specific signals were revealed after incubation with appropriate secondary antibodies (Mouse IgG Secondary Antibody, Thermo Fisher Scientific, Waltham, MA, USA, 1.5 μg/μl, 1:2000 diluted, or ExoAb Rabbit Secondary Antibody System Biosciences, Palo Alto, CA, USA, dilution 1:20,000), followed by detection with ECL Westar Supernova (Cyanagen, Bologna, Italy). Images were acquired with ChemiDoc Imaging System (Bio-Rad, Milan, Italy).

#### Confocal laser scanning microscopy

As a proof of concept of EV internalization in our system, EV deriving from ASC^GFP+^ [41] (kindly provided by Dr. Giulio Alessandri of IRCCS Neurological Institute Carlo Besta, Milan) were isolated through standard procedures and administered in vitro to CH seeded on glass coverslips. After 3 days, specimens were fixed in 4% paraformaldehyde, permeabilized with 0.1% Triton X-100, and incubated overnight at 4 °C with a monoclonal antibody raised against β-Tubulin (Sigma-Aldrich, St. Louis, MO, USA, 2 mg/ml, 1:100 diluted). The next day, samples were incubated at room temperature for 45 min with a goat anti-mouse secondary antibody conjugated with Alexa Fluor® 568 (Abcam, Cambridge, UK, 2 mg/ml, 1:1000 diluted). After 3 washes, coverslips were then mounted using ProLong™ Diamond AntifadeMountant with DAPI (Thermo Fisher Scientific, Waltham, MA, USA) and analyzed by the confocal laser scanning microscope TCS SP8 (Leica Microsystems CMS GmbH, Wetzlar, Germany) using a × 63 objective. The obtained images were processed with Las X (Leica Microsystems CMS GmbH, Wetzlar, Germany) and analyzed with Fiji software.

### In vitro OA induction and treatments

In our experimental set up, CH were always employed at 1st culture passage in order to prevent their de-differentiation [42]. We clarified this aspect in the text following the reviewer’s suggestion. Briefly, CH were seeded at the density of 10^4^ cells/cm^2^ in tissue culture-treated 6-well plates (Corning Incorporated, Corning, NY, USA) and cultured in complete medium until the full confluence was reached [43], then shifted in a complete medium containing 1% FBS, treated with 10 ng/ml TNFα to mimic OA microenvironment [27, 44] and CM (38.7 ± 16 μl) or EV (6.8 ± 2.2 μl) from 5 × 10^5^ ASC. OA was induced by TNFα for 3 and 6 days, concurrently with CM or EV treatment. At day 3 or 6, without any media change, supernatants were collected and cells lysed for further analyses.

### Western blotting of CH samples

CH were lysed in 50 mM Tris-HCl (pH 7.5), 150 mM NaCl, 1% NP-40, and 0.1% SDS supplemented with protease inhibitor cocktail (PIC) and 2 mM PMSF. Upon incubation on ice for 30 min, lysates were centrifuged for 15 min at 15,000×*g*, 4 °C, in order to eliminate cell membranes and collect the cytosolic fraction. The protein content of each sample was quantified through BCA Assay (Thermo Fisher Scientific, Waltham, MA, USA). Measurements were performed in technical duplicates. Samples were analyzed by 10% SDS-PAGE and Western blotting (WB), using standard protocols [27]. For each sample, 10 μg of protein extract were loaded and probed with the following primary antibodies: rabbit anti-Collagen X (Thermo Fisher Scientific, Waltham, MA, USA, dilution 1:100), mouse anti-MMP13 (Thermo Fisher Scientific, Waltham, MA, USA, 0.4 μg/μl, 1:100 diluted), rabbit anti-MMP3 (Cell Signaling, Danvers, MA, USA, dilution 1:1000), rabbit anti-Connexin 43 (Cell Signaling, Danvers, MA, USA, dilution 1:1000), and goat anti-GAPDH (Santa Cruz Biotechnology, 0.1 μg/μl, 1:1000 diluted). Specific bands were revealed upon incubation with appropriate secondary antibodies conjugated to horseradish peroxidase (Rabbit IgG Secondary antibody, Thermo Fisher Scientific, Waltham, MA, USA, dilution 1:10,000; Mouse IgG Secondary Antibody, Thermo Fisher Scientific, Waltham, MA, USA, dilution 1:6000; Goat IgG Secondary Antibody, Santa Cruz Biotechnology, CA, USA; 0.1 μg/μl, 1:6000 diluted) followed by detection with ECL Westar Supernova (Cyanagen, Bologna, Italy). After image acquisition with ChemiDoc Imaging System, protein expression was quantified through Image Lab Software (Bio-Rad, Milan, Italy). To normalize target protein expression, the band intensity of each sample was divided by the intensity of the loading control protein GAPDH. Then, the fold change was calculated by dividing the normalized expression from each lane by the normalized expression of the control sample (CTRL = 1).

### Analyses of culture supernatants

CH culture supernatants were collected and centrifuged for 5 min at 2000×*g*, 4 °C, to remove dead cells and debris, aliquoted and stored at − 20 °C. MMP activity was assessed with SensoLyte 520 Generic MMP Activity Kit (AnaSpec, Fremont, CA, USA), following the standard protocols. Briefly, pro-enzyme activation was performed through incubation with 1mM AMPA (4-aminophenylmercuric acetate) for 3 h at 37 °C in order to assess simultaneously the activity of different MMP. Measurements were performed in technical duplicates. The length of this incubation was chosen according to the manufacturer’s instructions as the preferential activation time to assess the activity of MMP-1 and -3, both strongly involved in OA. Samples were then incubated with the appropriate substrate for 45 min to run the enzymatic reaction and the resulting fluorescence signal (excitation *λ* = 490 nm, emission *λ* = 520 nm) was read with Wallac Victor II (Perkin Elmer, Milan, Italy). ADAMTS4 activity was tested using SensoLyte 520 Aggrecanase-1 Assay Kit (AnaSpec, Fremont, CA, USA), following standard procedures. PGE2 levels were assessed through Prostaglandin E2 Human Competitive ELISA Kit (Thermo Fisher Scientific, Waltham, MA, USA) following the kit instructions; then, data were analyzed with MyAssays analysis tool (https://www.myassays.com). Measurements were performed in technical duplicates.

### nLC-MS/MS of ASC-CM and -EV

ASC-CM and ASC-EV samples were analyzed by differential proteomics. Twenty micrograms of total proteins from each sample were in-solution digested using filter-aided sample preparation (FASP) protocol, as reported in literature [45]. Aliquots of the samples containing tryptic peptides were desalted using StageTip C18 (Thermo Fisher Scientific, Bremen, Germany) and analyzed by nLC-MS/MS using a Q-Exactive mass spectrometer (Thermo Fisher Scientific, Bremen, Germany) equipped with a nano-electrospray ion source (Proxeon Biosystems, Odense, Denmark) and a nUPLC Easy nLC 1000 (Proxeon Biosystems, Odense, Denmark). Peptide separations occurred on a homemade (75 μm i.d., 15 cm long) reverse phase silica capillary column, packed with 1.9-μm ReproSil-Pur 120 C18-AQ (Dr. Maisch HPLC GmbH, Ammerbuch-Entringen, Germany). A gradient of eluents A (distilled water with 0.1% v/v formic acid) and B (acetonitrile with 0.1% v/v formic acid) was used to achieve separation (300 nL/min flow rate). After 5 min at 2% of B, the acetonitrile phase was increased up to 40% B in 83 min, followed by a wash step at 90% of B. Full scan spectra were acquired with the lock-mass option, resolution set to 70,000 and mass range from m/z 300 to 2000 Da. The ten most intense doubly and triply charged ions were selected and fragmented. All MS/MS samples were analyzed using Mascot (version 2.6, Matrix Science) search engine to search the human_proteome 20190703 (96,470 sequences; 38,319,731 residues). Searches were performed with the following settings: trypsin as proteolytic enzyme, 2 missed cleavages allowed, carbamidomethylation on cysteine as fixed modification, protein N-terminus-acetylation and methionine oxidation as variable modifications, and mass tolerance was set to 5 ppm and to 0.02 Da for precursor and fragment ions, respectively. To quantify proteins, the raw data were loaded into the MaxQuant [46] software version 1.6.1.0. Label-free protein quantification was based on the intensities of precursors. The experiments were performed in technical triplicates. Data are expressed as label-free quantification (LFQ) intensity, count per second (cps). In order to identify differences between ASC-CM and -EV that can be relevant in the OA context, the list of proteins quantified by nLC-MS/MS was run using the following keywords: Chondro-, Metabol-, Catabol-, Inflamm-, and Matrix. The keywords were chosen considering that OA is an inflammatory disease affecting the osteochondral unit and altering extracellular matrix metabolism and catabolism. The functional enriched processes were then identified using STRING (Search Tool for the Retrieval of Interacting Genes/Proteins) (https://string-db.org/). Each identified process is reported in Supplementary Table 2 along with its GO identifier, the number of mapped genes within our dataset, the number of mapped genes in the reference dataset, its *p* value, and the list of gene names within our dataset. Using the gene names assigned to each of the five selected functional processes and their protein abundance levels measured by nLC-MS/MS, principal component analysis (PCA), and heat maps were obtained using XLSTAT software. To validate nLC-MS/MS data, the presence of selected molecules in ASC secretome was confirmed by immunoassays through the Bio-Plex Multiplex System (Bio-Rad, Milan, Italy). In details, OPG and DKK-1 were quantified with the Human Bone Magnetic Bead Panel-Bone Metabolism Multiplex Assay (HBNMAG-51K, Millipore, Burlington, MA, USA), MMP1 and 2 with the Human MMP Magnetic Bead Panel 2 (HMMP2MAG-55 K, Millipore, Burlington, MA, USA), while TIMP-1,- 2 and -3 with the Human TIMP Magnetic Luminex Performance Assay (LKTM003, R&D Systems, Minneapolis, MN, USA). Technical duplicates were analyzed for each CM sample following previously described procedures [27, 47] and data analysis was performed with the MAGPIX xPONENT 4.2 software (Luminex Corporation, Austin, TX, USA). The concentration of the selected molecules is reported in Supplementary Table 3 along with the processes in which each factor is involved, as indicated in Supplementary Table 2.

### Statistics

Statistical analysis was performed by one-way analysis of variance (ANOVA) using Tukey’s post hoc test in case of normally distributed measures, otherwise (i.e., PGE2 and COL10A1 data) by Friedman’s test followed by Dunn’s multiple comparison. Differences were considered significant at *p* ≤ 0.05. Unless otherwise stated, data are expressed as mean ± SD of 5–7 independent experiments. All the analyses were performed using Prism 5 (GraphPad Software, La Jolla, CA, USA).

## Results

### ASC-CM and -EV characterization

NTA demonstrates a comparable size distribution between ASC-CM and ASC-EV samples (Fig. 1a), with the 50% of events falling inside the dimensional range of 147 ± 14 and 133 ± 19 nm, respectively. Given the same number of donor ASC, the two preparations differ for particle concentration, with a post-ultracentrifugation recovery of about 30% of the input (ASC-CM = 9.0 ± 4.3 × 10^8^ particles/10^6^cells and ASC-EV = 2.6 ± 0.9 × 10^8^ particles/10^6^cells). Flow cytometry confirms a similar EV size (Fig. 1b) and a comparable expression of vesicular markers (Fig. 1c–e). In details, most of the vesicles are included within 240 nm (Fig. 1b) and the percentage of positive events for the EV markers CD63 (> 79%), CD81 (> 76%), and CD9 (> 28%) is alike in ASC-CM and -EV (Fig. 1c–e). Transmission electron microscopy supported the presence of nanoparticles with the characteristic cup-shaped morphology and the expected size range (Fig. 1f). Furthermore, protein expression of HSP70, Flotillin-1 (FLOT1), and CD9 shows a similar vesicular phenotype (Fig. 1g). ASC-EV were able to interact with recipient cells: indeed in Fig. 1h, as a proof of concept, the incorporation of EV derived by ASC^GFP+^ [41] in CH is shown. The spatial co-localization of the green (EV or EV cluster from ASC^GFP+^) and the red (cytoskeleton) signals shown in the orthogonal views suggests the incorporation of vesicular elements in the recipient cell. Additional evidence is provided in Supplementary Figure 1. At last, ASC-CM contains 25-fold more proteins (in terms of quantity) than EV samples, indicating that soluble factors are also abundant (Fig. 1i, l).

### ASC-CM, but not ASC-EV, significantly reduces TNFα-induced MMP activity

**Fig. 2:**
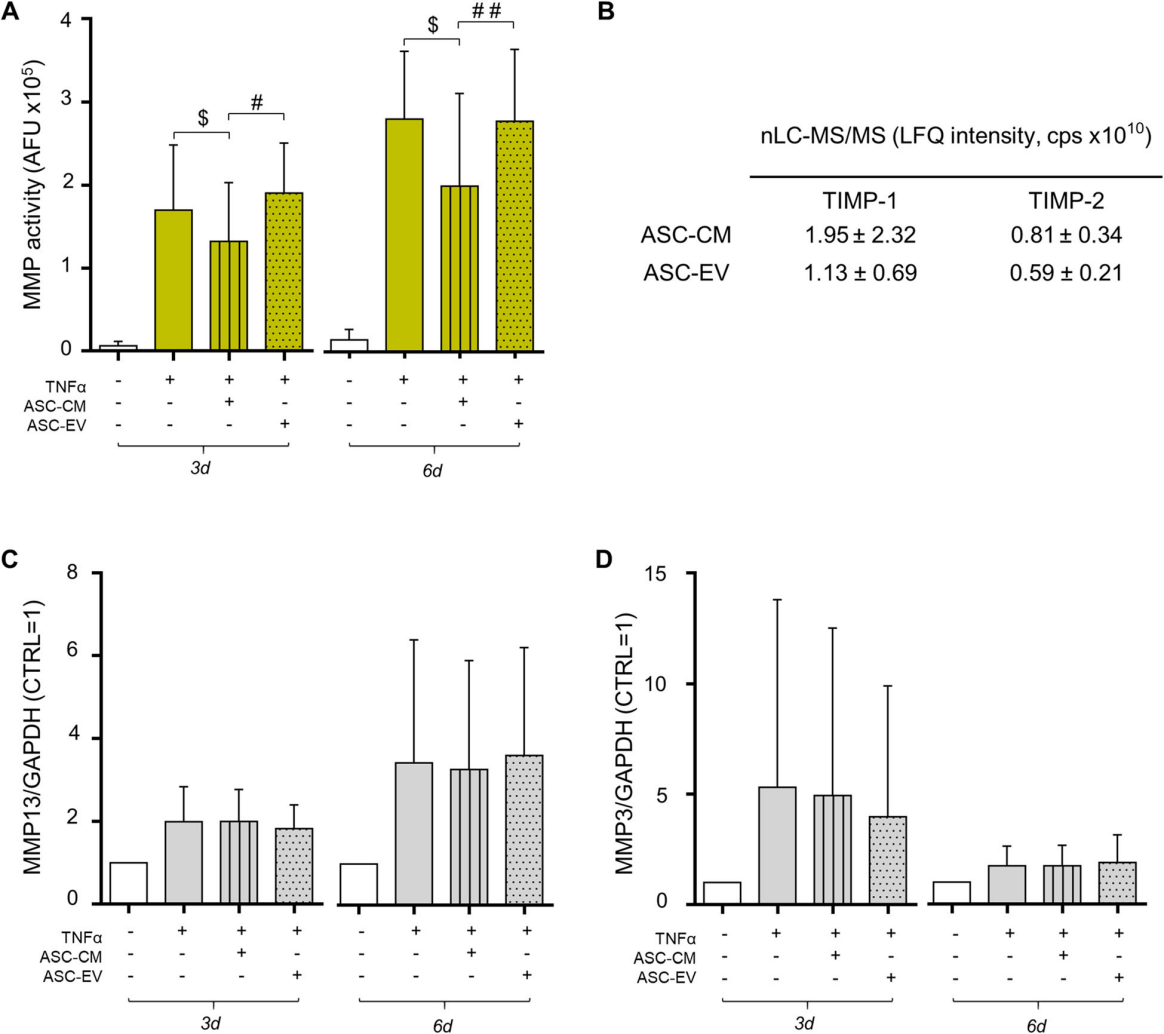
Reduction of MMP activity by ASC-CM, TIMP quantification, and MMP expression. **a** MMP activity, analyzed in CH culture medium (*n* = 7 independent experiments) 3 and 6 days after the treatments, is expressed as arbitrary fluorescence units (AFU). All conditions statistically differ from control (at day 3: TNF *p* < .01, TNF+ASC-CM *p* < .05, and TNF+ASC-EV *p* < .001; at day 6: TNF *p* < .001, TNF+ASC-CM *p* < .05, and TNF+ASC-EV *p* < .001). Significance vs 10 ng/ml TNFα is shown as ^$^*p* < .05; vs ASC-EV as ^#^*p* < .05, ##*p* < .01. **b** TIMP-1 and 2 data are expressed as label-free quantification (LFQ) intensity, count per second (cps) from differential proteomic analysis of 20 μg of ASC-CM and -EV proteins. Means ± SD (*n* = 3) are shown. **c**, **d** Quantification of the expression of MMP-13 (**c**) and MMP-3 (**d**) in TNFα-stimulated and ASC-CM- or -EV-treated CH at day 3 and 6 analyzed by Western blot. Data (*n* = 5 independent experiments) were normalized on GAPDH and expressed as relative values (CTRL = 1)

Unstimulated CH secrete low levels of active matrix metalloproteinases (MMP), but their activity is strongly increased by the inflammatory stimulus at both time points (+ 2498% and + 1781%, respectively) (Fig. 2a). ASC-CM significantly reverts TNFα-induced activation of about 22% and 29% at day 3 and 6 (Fig. 2a, left and right panel, respectively). In contrast, no effect was exerted by the treatment with EV. Differential proteomics allowed the quantification of TIMP-1 and -2, the two most abundant TIMP (tissue inhibitors of MMP) in ASC secretome [24, 27] (Fig. 2b). These data, obtained analyzing the same amount of proteins for ASC-CM and -EV, revealed that both inhibitors are slightly more represented in CM samples (Fig. 2b). Actually, CM contains far more TIMP than EV per ASC number, since EV samples are derived from a 25-fold higher number of cells. The fact that CM acts mainly through the presence of MMP inhibitors is further supported by the lack of effect on MMP expression. Protein expression of MMP-13 and MMP-3, two matrix-degrading enzymes involved in OA [48–50], is displayed in Fig. 2c and d and in Supplementary Figure 2. Despite the large inter-donor variability due to the use of patient-derived articular chondrocytes, a clear effect of TNFα on MMP expression is always present. Differently, CM exerts no effect on their overexpression, as previously shown [27], nor did EV. ADAMTS-4 activity was also tested, since TIMP act also on aggrecanases. However, in our experimental setting, its activity was always undetectable (data not shown).

### ASC-CM and ASC-EV differently modulate inflammation and hypertrophy markers in TNFα-treated CH

**Fig. 3:**
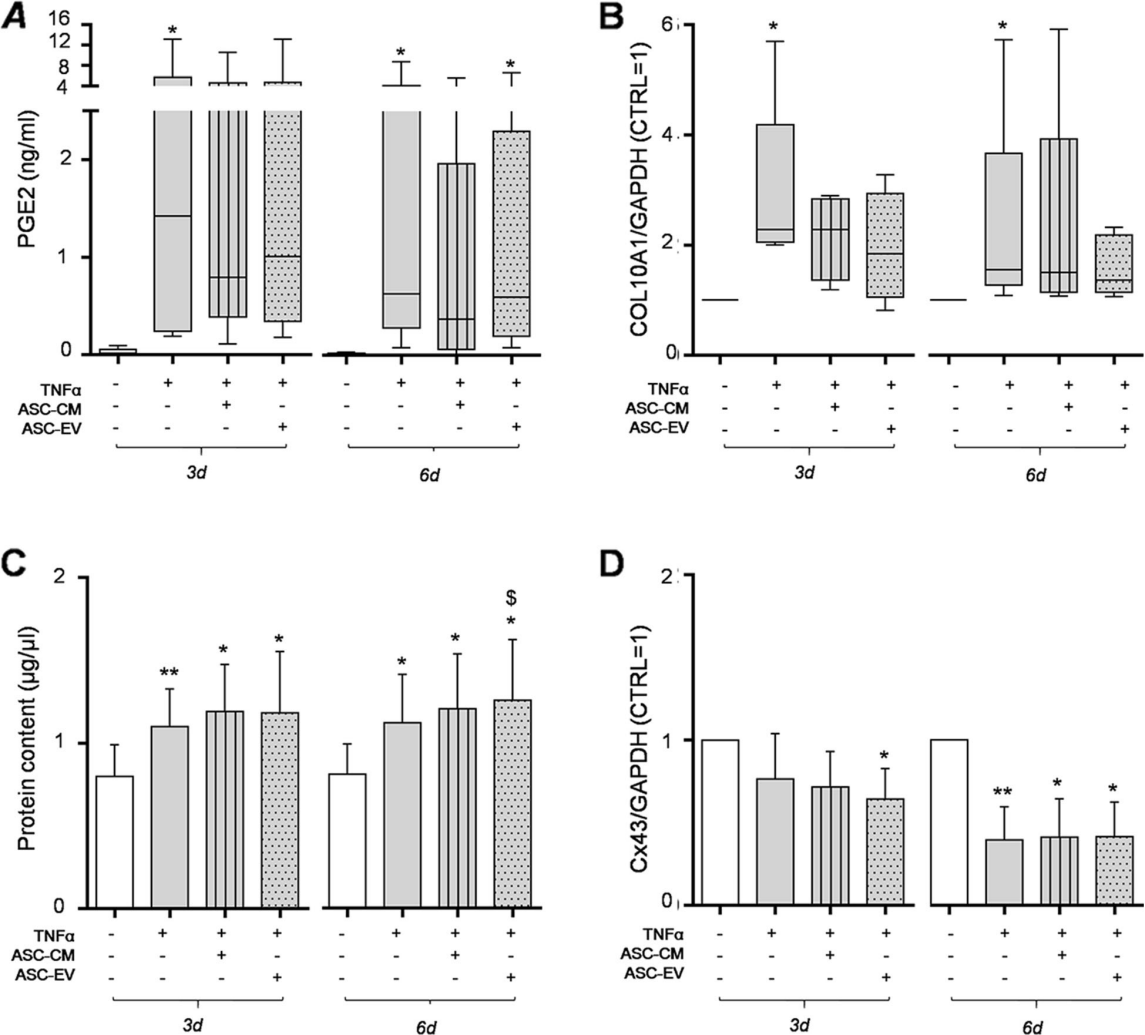
Hypertrophy and inflammatory markers induced by TNFα treatment. **a** PGE2 levels, quantified in CH culture medium at day 3 and 6 after treatments, are expressed as ng/ml (*n* = 6 independent experiments). **b** COL10A1 expression by Western blot analysis. Data (*n* = 5 independent experiments) were normalized on GAPDH and shown as relative values (CTRL = 1). Data in **a** and **b** were analyzed by Friedman’s test followed by Dunn’s multiple comparison test and significance vs CTRL is shown as *p < .05. For each column, the box extends from the 25th to 75th percentiles, the line in the middle is plotted at the median while the whiskers indicate minimum and maximum value. **c** CH protein concentration at day 3 and 6 (left and right panel, respectively). Significance vs CTRL is shown as *p < .05 and **p < .01; vs 10 ng/ml TNFα as ^$^*p* < .05 (*n* = 7 independent experiments). **d** Cx43 expression by Western blot. Data (*n* = 5 independent experiments) were normalized on GAPDH and expressed as relative values (CTRL = 1). Significance vs CTRL is shown as *p < .05 and **p < .01

In order to investigate CH activation by TNFα and an effect of ASC secretome, we examined PGE2 release, total protein content, Collagen X, and Connexin 43 expression. As expected, TNFα raises the extracellular concentration of the inflammatory mediator PGE2, scaling it more than 2 orders of magnitude at both time points (Fig. 3a). Since high PGE2 concentrations can inhibit proteoglycan synthesis and stimulate matrix degradation [51, 52], a possible counteracting effect of ASC secretome was hypothesized. ASC-CM decreased PGE2 upregulation up to 40% at day 6 (Fig. 3a, right panel). Conversely, ASC-EV did not affect TNFα-induced PGE2 levels.

By contrast, both ASC-derived treatments partly blunt TNFα effect (about − 30%) on the production of Collagen type X (COL10A1, Fig. 3b), a short chain collagen expressed by hypertrophic CH [50]. EV induced a more long-lasting effect while ASC-CM acted incisively only at the early time point (Fig. 3b, right and left panel, respectively, Supplementary Figure 2).

The TNFα-induced phenotypic shift towards hypertrophy can be inferred by the significant increase in CH total protein content (Fig. 3c, + 38%), due to an increase in cell proliferation. ASC-CM did not counteract TNFα-induced cell growth, while, at day 6, ASC-EV act in synergy with the inflammatory cytokine, fostering its pro-proliferative action (+ 55% vs CTRL, + 12% vs TNFα alone) (Fig. 3c, right panel).

At last, the expression of Connexin 43 (Cx43), the most widely expressed connexin in the musculoskeletal system [53, 54], was investigated. Its levels are clearly down-modulated by TNFα (Fig. 3d, Supplementary Figure 2), especially in the long run (− 60% vs CTRL, Fig. 3d right panel). ASC-EV further reduced Cx43 expression at day 3 (− 16% vs TNFα alone, Fig. 3d left panel) while the effect of ASC-CM is negligible.

### ASC-CM and ASC-EV present different factors of interest in the OA context

**Fig. 4:**
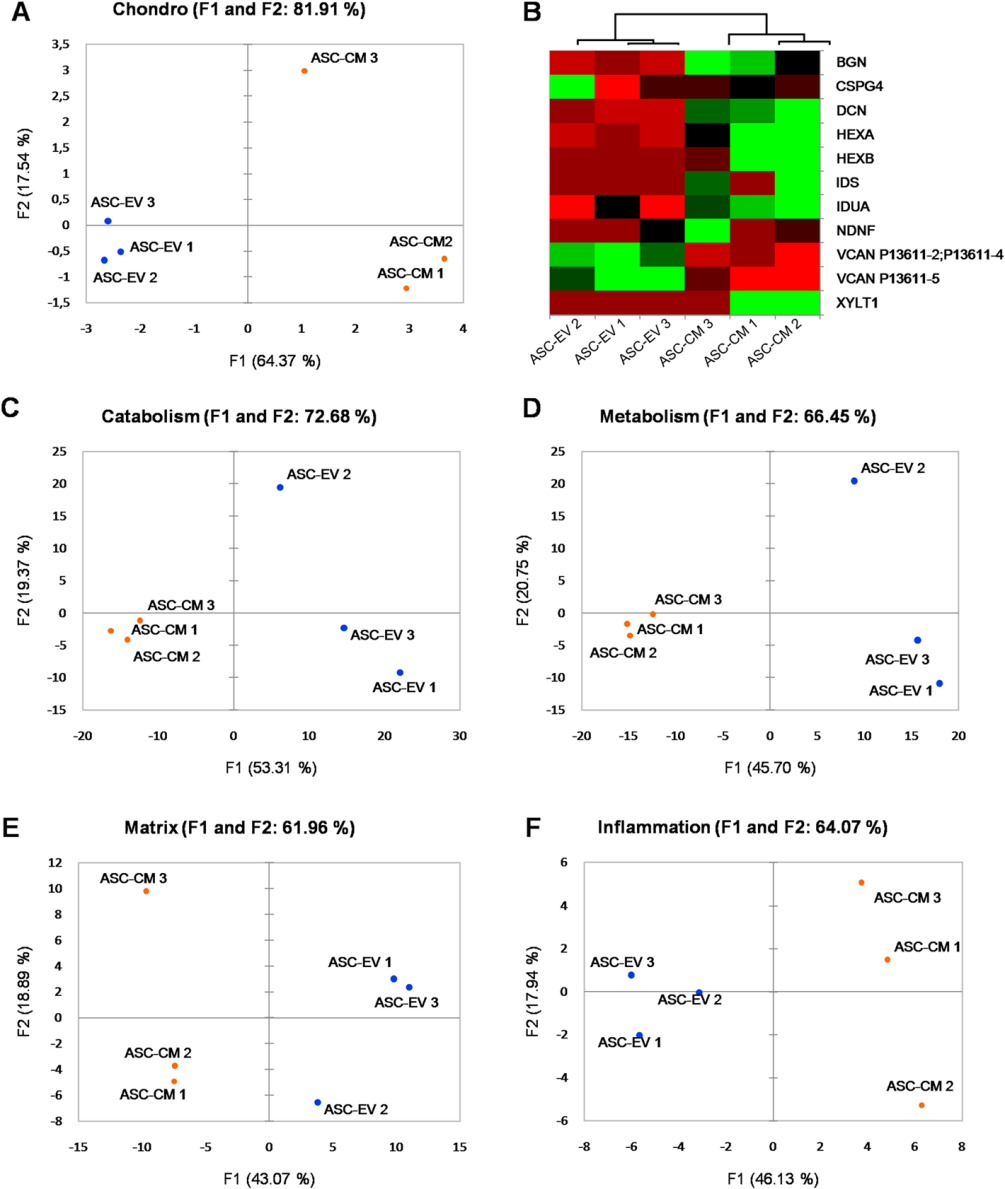
PCA and heat map of chondroitin sulfate-related factors. PCA plots of the samples based on 10 chondroitin sulfate- (**a**), 56 inflammation- (**c**), 425 catabolism- (**d**), 459 metabolism- (**e**), and 169 matrix- (**f**) mapped gene names. Color scale: red (down-represented) to green (up-represented) through black. (**b**) Heat map for the chondroitin sulfate process-associated genes, constructed on the basis of protein abundance levels estimated by nLC-MS/MS in 3 ASC-CM (ASC-CM 1-3) and 3 ASC-EV (ASC-EV 1-3) samples. Principal component analysis (PCA) and heat maps were obtained using XLSTAT software. F1 and F2, factor 1 and 2

Proteomic data analysis through OA-related keywords confirms that CM and EV protein profiles are distinct for all the considered processes (Fig. 4 and Supplementary Table 4). Of note, the analysis on chondroitin sulfate factors led to an important discrimination between CM and EV, with more than 81.9% of variance explained by factors 1 and 2 (Fig. 4a, Supplementary Table 4). The most relevant differences were distinguishable in the expression of Versican (greater than in ASC-EV) and Decorin and Biglycan (greater than in ASC-CM) (Fig. 4b and Supplementary Table 5). At last, a manual check for other proteins linked to OA allowed the identification of Bone Morphogenetic Protein 1, Dickkopf-related protein 3, and 4 members of the ADAM (A Disintegrin and Metalloproteinase) family (in details ADAM10, ADAM12, ADAM17, and ADAM9) as more abundant in the CM samples (Supplementary Table 5). PCA on the factors associated to catabolism, metabolism, matrix, and inflammation allowed a distinction between CM and EV, with a score always higher than 60% of variance explained by F1 and F2 (Fig. 4c–f and Supplementary Table 4).

## Discussion

With the discovery that MSC engraftment and differentiation play a partial role in the success of cell therapy [55], over the years the scientific interest has shifted towards MSC-secreted factors. In the last decade, the number of studies focused on the physical and functional characterization of MSC secretome has grown exponentially. Recently some cell-free products have reached the clinics in phase I and II trials [56] for diverse applications in which the beneficial action of the cells of origin was already well-documented [20, 21, 57]: MSC-CM in wound healing [58], alopecia [59, 60], bone regeneration [61], and multiple sclerosis [62], while MSC-EV in graft versus host disease [63], chronic kidney disease [64], type 1 diabetes, macular holes, and acute ischemic stroke (cited in [65]). Considering future clinical applications in the OA management, here, we compared the potential the potential of ASC whole secretome versus its EV component. Our treatment strategy follows what right now represents the gold standard for cell therapy, i.e., cell number-based dosage. This approach was already reported in literature for EV administration, both in vitro [66] and in vivo [67]. At first, the assumption that ASC-CM preparation allows a complete retention of the vesicular components [27, 68] has been validated. Then, we compared the two preparations focusing on size distribution, the presence of EV markers [16, 40] and particle concentration. Our data confirm that the EV isolation procedure through ultracentrifugation neither affected the quality of the particles nor enriched any subpopulation. Indeed, we confirmed a similar vesicular profile between ASC-CM and -EV in terms of dimensions, antigen expression, and granularity/complexity. Moreover, we give evidence of a 3 times higher vesicular yield in concentrated CM samples compared to EV ones, due to particle loss during the ultracentrifugation procedure [69–71]. Therefore, our in vitro data compare two preparations deriving from the same number of donor cells, with CM accounting for both soluble factors and a higher number of retained particles in comparison to ultracentrifuge-isolated EV. Conversely, our differential proteomic analysis considers the same protein amount for ASC-CM and -EV (20 μg/sample).

Both in vitro evidence and the differential analysis of the protein content between the two preparations suggest a higher therapeutic anti-OA potential of ASC-CM over ASC-EV. One of the most relevant differences was the lack of inhibition of MMP activity by ASC-EV, confirming our previous assumption that the blunting of MMP activity is a direct consequence of active TIMP in ASC secretome [27]. Indeed, these inhibitors are more represented in the complete secretome compared to the vesicular fraction. To our knowledge, it is the first time that the greater abundance of freely dissolved TIMP rather than EV-released ones is clearly defined. This aspect gains relevance in the light of developing MMP inhibitors as potential pharmacological tools in the management of a variety of diseases [72]. It also points out that for every pathology implying the aberrant activation of MMP [73, 74], the complete secretome, rather than the EV fraction alone, should be considered the more promising therapeutic strategy.

In addition, ASC-CM was more efficient in reducing the release of the inflammatory mediator PGE2 by TNFα-stimulated CH. This prostaglandin is known to exert multiple opposed functions based on its concentration. In our case, the down-modulation of PGE2 aimed at restoring its physiologic levels linked to a healthy CH phenotype. Indeed unstimulated chondrocytes release low amount of PGE2 (average release of about 20 pg/ml) that are consistent with the concentration known to inhibit collagen cleavage and the expression of hypertrophy markers [75]. By contrast, TNFα raised PGE2 levels above the pro-anabolic concentration (average release of about 2.5 ng/ml) and it is known that similar levels (from 1 to 1000 ng/ml) exert a pro-catabolic and anti-anabolic effect on articular chondrocytes [52, 76]. As follows, the reduction induced by ASC-CM appears beneficial. Vonk et al. [77] observed a more marked effect of CM compared to EV in reducing TNFα downstream effectors by CH, in particular looking at cyclooxygenase-2 (COX2) expression. Indeed, TNFα treatment induces PGE2 release through the activation of COX2 transcription via NF-κB [78]. However, our data (Supplementary Figure 3) show the lack of a clear-cut effect of ASC-CM on TNFα-induced COX2 protein expression at 3 and 6 days, suggesting that the mechanisms underlying the blunting of PGE2 may act at a different level. Further investigations are currently ongoing to elucidate this aspect.

In our opinion the observed minor ASC-EV effects cannot be ascribed to the lack of internalization of EV into recipient cells. Indeed, administering ASC^GFP+^-EV to stained CH, a clear intracellular co-localization of the fluorescent signals was observed, suggesting an efficient EV uptake. ASC-EV incorporation has been already reported in other in vitro systems [39, 66] and future investigations will be necessary to disclose its underlying mechanisms (e.g., endocytosis and interaction of cell surface receptors [79]). Moreover, here, we show that EV modulate hypertrophy markers, contrasting TNFα action on COL10A1 expression to a similar—if not more marked—extent as ASC-CM. EV effect on chondrocytes seems less donor-dependent and more long-lasting compared to ASC-CM, suggesting that the mediator of COL10A1 reduction may be stored in EV and its beneficial action can be reduced by soluble factors. The regulation of COL10A1 gene expression during CH hypertrophic differentiation depends on multiple factors, including both transactivators and repressors (such as Runx2 and Sox9, respectively) and has not been fully elucidated yet [80]. However, in vitro evidences demonstrate that the overexpression of the miRNA hsa-miR-148a decreases COL10A1 levels together with two other OA-related genes, MMP13 and ADAMTS5 [81]. Of note, the presence of hsa-miR-148a in EV derived from both naïve and IFNγ-primed ASC has recently been demonstrated by Ragni et al., as shown in their Additional File 3 [82]. Moreover, here, we have identified Versican, a chondroitin sulfate proteoglycan, more abundant in EV. Interestingly, it plays important roles in chondrogenesis and in the retention of cartilage extracellular matrix (ECM) [83]. Even though EV reduced COL10A1 expression, suggesting a potential modulation of hypertrophy, no reduction on TNFα-mediated increased metabolism and proliferation was depicted. By contrast, we observed a slight increase in cell growth, previously reported also by Vonk et al. [77]. However, in their setting TNFα induced a reduction in chondrocyte proliferation that we have never observed in our in vitro model [27]. We ascribed the increase of chondrocyte proliferation by TNFα to the reduction of Cx43 expression, consistently with the fact that Cx43 C-terminal domain (CTD) influences chondrocyte proliferation and phenotype maintenance [54]. Moreover, Cx43 reduction by TNFα has been shown in several cell types and plausible causes could be the activation of ubiquitin-proteasome system [84] or the activation of JNK by TNFα [85]. Additionally, the cleavage of Cx43 CTD by MMP has been already documented [86] and further experiments will be performed with the aim of evaluating whether the reduced Cx43 signal depends either on the modulation of its protein expression or on a post-translational mechanism.

Taken together, these results show that, given the same number of donor cells, ASC-CM is more efficient than ultracentrifuge-isolated EV. ASC-CM and ASC-EV present different protein composition not only in terms of quantity but also in terms of quality. Indeed, PCA shows specific differences in OA-related processes, spanning from ECM maintenance to inflammation. In details, ASC-CM appears to be more abundant in cartilage protective factors. Besides TIMP, we have identified other proteins involved in ECM organization and chondrogenesis. Among them, Biglycan (BGN) and Decorin (DCN), two small leucine-rich proteoglycans (SLRP), can bind different types of collagen and organize the fibrils. Moreover, they interact with TGFβ1 and control its signaling [87]. Also, BMP-1 plays an important role in collagen fibril organization. It cleaves C-propeptides of procollagens I, II, and III allowing the correct incorporation of monomers into growing fibrils [88]. Furthermore, it activates diverse TGFβ superfamily proteins that are fundamental for ECM formation and growth factor activity [89]. DKK-3 exerts protective roles in OA cartilage by interacting with TGFβ signaling, too [90]. Considering that, in the OA context, both collagen fibril organization and TGFβ signaling are affected [91, 92], the soluble factors present in ASC-CM could help in re-establishing a physiological condition. ASC-CM is enriched also in four ADAM, a group of enzymes involved in joint homeostasis. ADAM9 is involved in chondrogenesis [93], ADAM10 has a controversial functionality and its expression is increased in both developing and OA cartilage. Since its substrate in the articular district has not been identified yet, its implications in pathological conditions are still unclear [94]. In addition, ADAM12 seems to be associated to OA and chondrocyte maturation [94]. The presence in ASC-CM of TNFα-activating pro-inflammatory ADAM17 could be dangerous; however, both its inhibitors TIMP-3 and CD9 [95] are present in the secretome as well [27], suggesting that this enzyme could be inactivated. This consideration highlights the complexity of secretome and the relevance of the balance between all the different factors.

## Conclusions

In conclusion, our study suggests that soluble and EV-associated factors concur in a strongly synergistic manner to the anti-inflammatory, anti-hypertrophic, and anti-catabolic effect of ASC secretome. Even though we mainly investigated the differences in terms of protein content between the two cell products, we need to consider that they surely differ also in terms of nucleic acids such as miRNA and lipid composition. To date, the knowledge of the bioactive components eliciting a therapeutic action in different preclinical scenarios is still very limited, and their characterization in the perspective of a clinical translation is still ongoing. However, CM preparations, compared to EV isolated by ultracentrifugation, present several advantages, such as a higher vesicular yield and a lower manipulation, accounting for an easier compliance with good manufacturing practices (GMP) and a better scalability. Indeed, from a clinical point of view, CM production is cheaper and faster than EV isolation by the currently available, routinely used, techniques (e.g., ultracentrifugation and size exclusion chromatography). Moreover, CM yields a wider array of bioactive factors, soluble, freely dissolved proteins, nucleic acids, and lipids, with respect to the vesicular fraction alone [66]. In the future, more complex in vitro models, such as organoids or models considering the interaction with other articular cell types (e.g., synoviocytes and osteoblasts), should be set and additional preclinical investigations, focused on the optimization of CM production, will be performed in order to confirm the efficacy of ASC-CM in the OA context.

## Supplementary Information

**Additional file 1: Supplementary Figure 1**. Representative images of EV incorporation by CH. EV derived from ASC^GFP+^ are indicated by green arrows, β-Tubulin was revealed with an Alexa Fluor® 568 conjugated antibody (red) and nuclei were stained with DAPI (blue). The scale bars indicate 10 μm and the orthogonal views referred to the EV encircled in yellow were obtained by Fiji software.

**Additional file 2: Supplementary Figure 2**. Representative Western Blot membrane for COL10A1, MMP-13, MMP-3, Cx43 and GAPDH.

**Additional file 3: Supplementary Figure 3**. (A) Quantification of the expression of COX2 (mAb #12282, Cell Signaling Technology, Danvers, MA, USA) in TNFα-stimulated and ASC-CM or -EV treated CH at day 3 and 6 analyzed by Western Blot. Data (*n* = 3 independent experiments) were normalized on GAPDH and expressed as relative values (CTRL = 1).(B) Representative Western Blot membrane for COX2 and GAPDH.

**Additional file 4: Supplementary Table 1**. ASC features. Details of culture conditions (CTRL, Osteoinduction and Adipoinduction) and performed assays are reported. **Supplementary Table 2**. Functional enriched processes identified with STRING through OA-related keywords (Chondro-, Metabol-,Catabol-, Inflamm- and Matrix). GO: Gene ontology identifier. **Supplementary Table 3**.Quantification of OA-related factors in ASC-CM (n = 3) expressed as mean ± SD. Details of the biological processes in which each factors is involved, according to Supplementary Table 2, are also provided. **Supplementary Table 4**. Principal Component Analysis (PCA) details by XLSTAT software. Number of factors analyzed for each OA-related keyword are reported in brackets. **Supplementary Table 5**. nLC-MS/MS quantification of OA-related factors in ASC-CM and ASC-EV (n = 3). Factors significantly different between CM and EV samples are in bold. Proteins more abundant in EV that in CM are highlighted in gray.

## Data Availability

The datasets used and/or analyzed during the current study are available from the corresponding author on reasonable request.

## Abbreviations

ACI: Autologous chondrocyte implantation
ADAM: A disintegrin and metalloproteinase
AMPA: 4-Aminophenylmercuric acetate
ASC: Adipose-derived stem/stromal cells
BGN: Biglycan
BMC: Bone marrow concentrate
BMI: Body mass index
CFSE: Carboxyfluorescein diacetate succinimidyl ester
CH: Articular chondrocytes
CM: Conditioned medium
COL10A1: Collagen type X
COX2: Cyclooxygenase-2
cps: Count per second
CTD: C-terminal domain
Cx43: Connexin 43
DCN: Decorin
DMOAD: Disease-modifying anti-OA drugs
ECM: Extracellular matrix
EV: Extracellular vesicles
FASP: Filter-aided sample preparation
GMP: Good manufacturing practices
LFQ: Label-free quantification
MMP: Matrix metalloproteinases
MSC: Mesenchymal stem/stromal cells
NSAID: Non-steroidal anti-inflammatory drugs
NTA: Nanoparticle tracking analysis
OA: Osteoarthritis
PCA: Principal component analysis
PIC: Protease inhibitor cocktail
PRP: Platelet-rich plasma
SLRP: Small leucine-rich proteoglycans
STRING: Search Tool for the Retrieval of Interacting Genes/Proteins
TIMP: Tissue inhibitors of matrix metalloproteinases
WB: Western blotting
