# Supplementary figures and images for: Comparison of two ASC-derived therapeutics in an in vitro OA model: secretome versus extracellular vesicles
Source: Stem Cell Res Ther. 2020 Dec 3;11:521. doi: 10.1186/s13287-020-02035-5 (PMC7711257; doi:10.1186/s13287-020-02035-5)

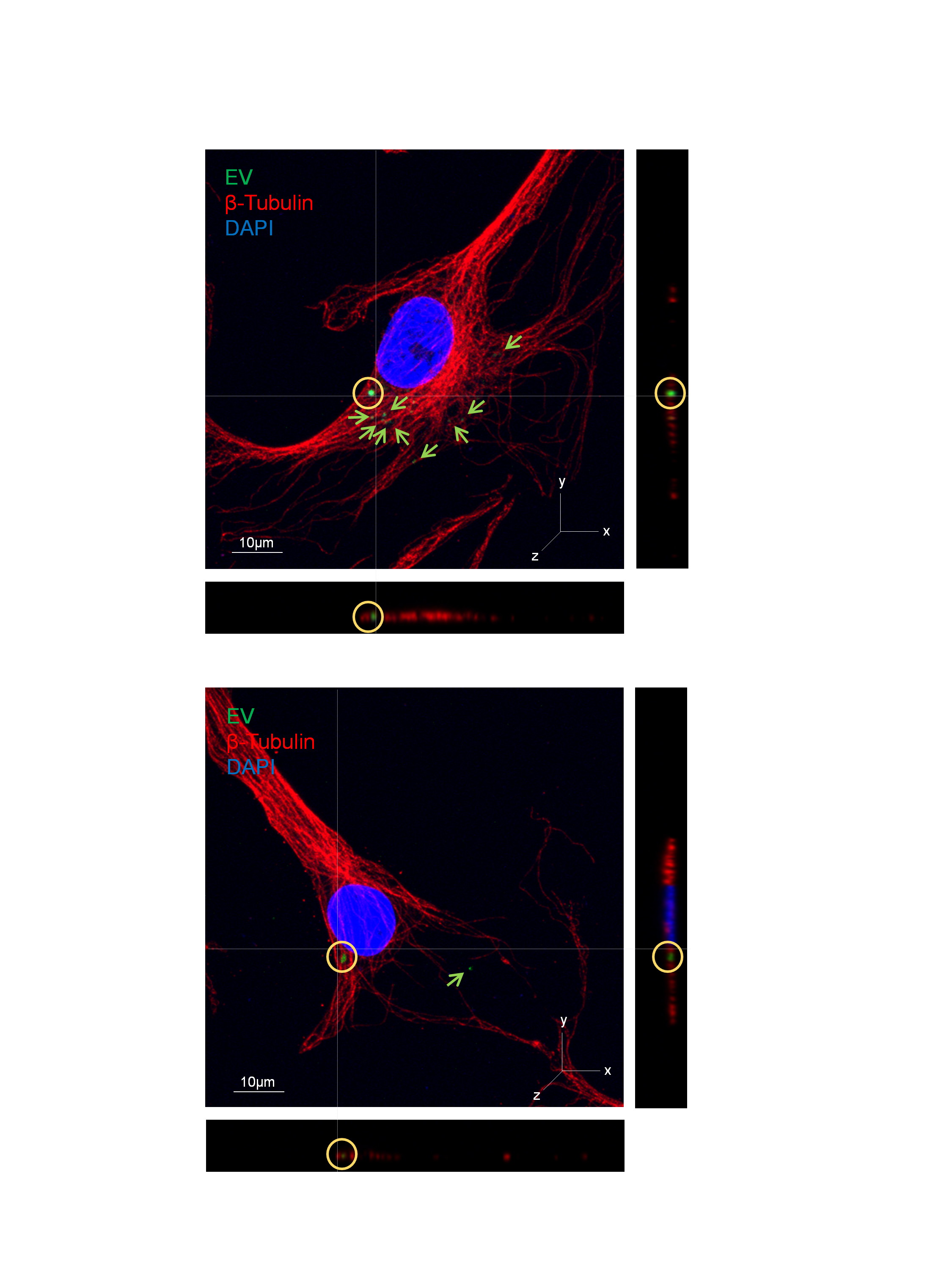

Supplement: Supplementary file 1 — Additional file 1: Supplementary Figure 1. Representative images of EV incorporation by CH. EV derived from ASCGFP+ are indicated by green arrows, β-Tubulin was revealed with an Alexa Fluor® 568 conjugated antibody (red) and nuclei were stained with DAPI (blue). The scale bars indicate 10 μm and the orthogonal views referred to the EV encircled in yellow were obtained by Fiji software. [file 13287_2020_2035_MOESM1_ESM.tif]

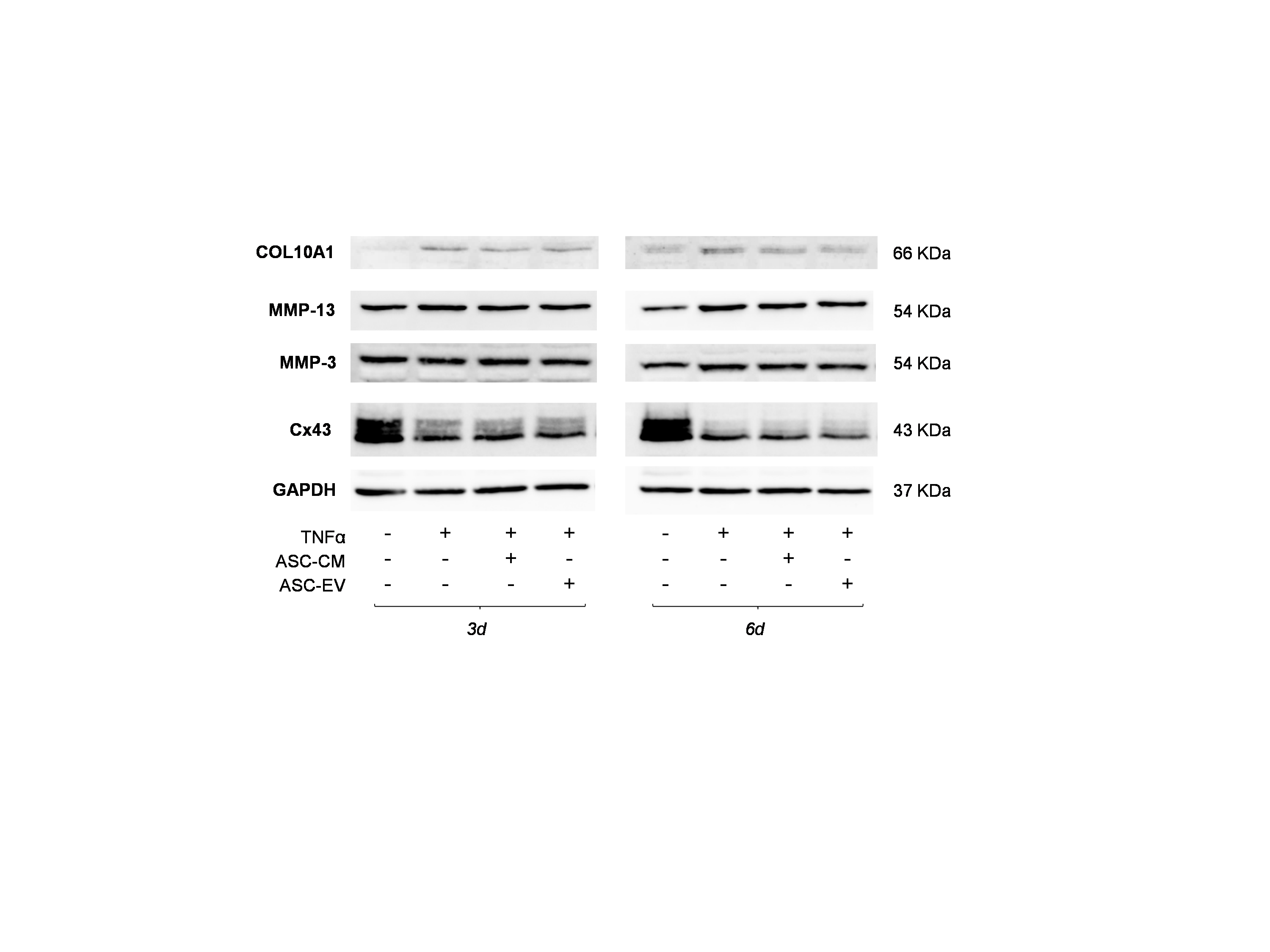

Supplement: Supplementary file 2 — Additional file 2: Supplementary Figure 2. Representative Western Blot membrane for COL10A1, MMP-13, MMP-3, Cx43 and GAPDH. [file 13287_2020_2035_MOESM2_ESM.tiff]

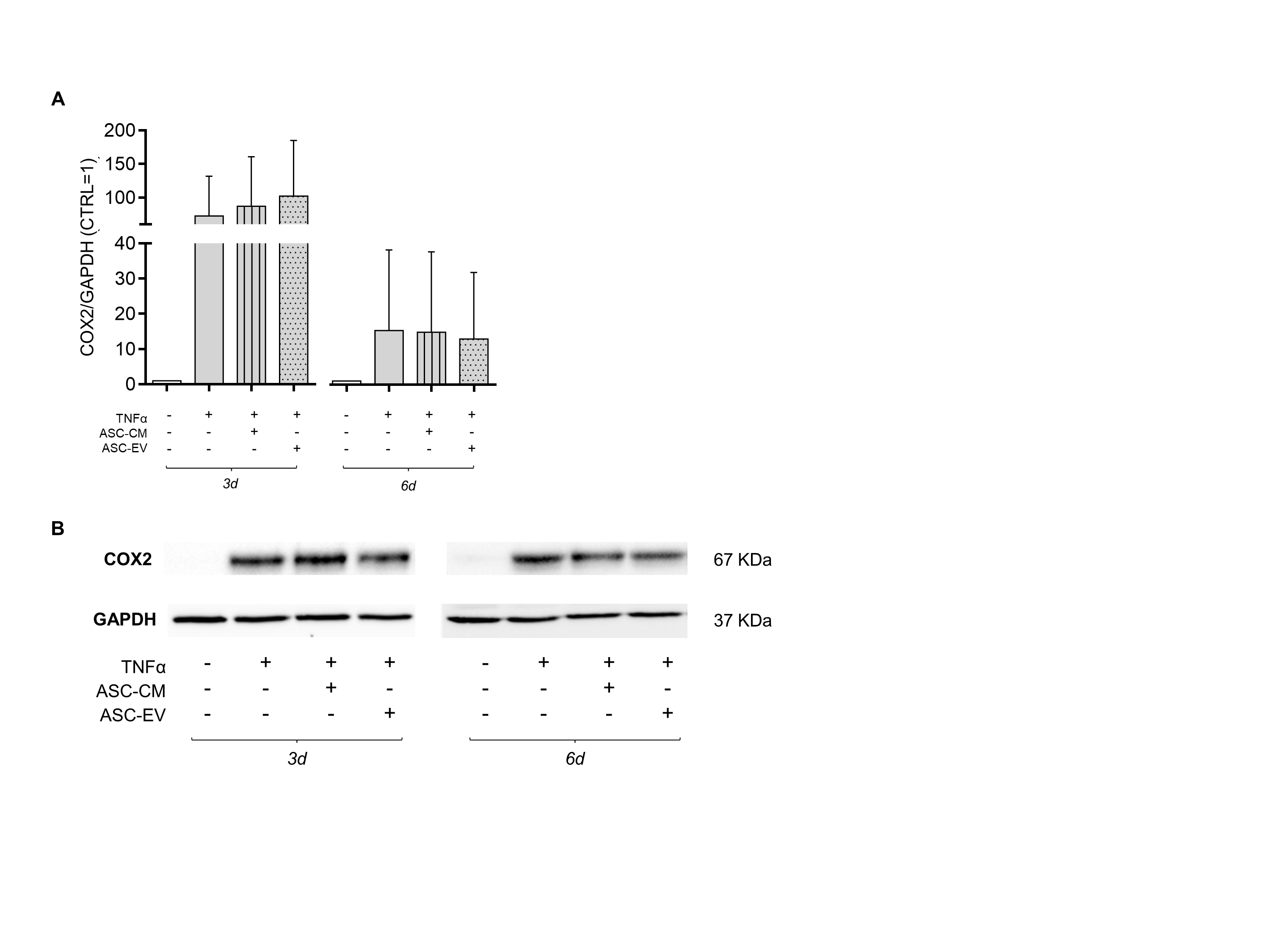

Supplement: Supplementary file 3 — Additional file 3: Supplementary Figure 3. (A) Quantification of the expression of COX2 (mAb #12282, Cell Signaling Technology, Danvers, MA, USA) in TNFα-stimulated and ASC-CM or -EV treated CH at day 3 and 6 analyzed by Western Blot. Data (n = 3 independent experiments) were normalized on GAPDH and expressed as relative values (CTRL = 1).(B) Representative Western Blot membrane for COX2 and GAPDH. [file 13287_2020_2035_MOESM3_ESM.tif]
